# Oxidized Calmodulin Kinase II Regulates Conduction Following Myocardial Infarction: A Computational Analysis

**DOI:** 10.1371/journal.pcbi.1000583

**Published:** 2009-12-04

**Authors:** Matthew D. Christensen, Wen Dun, Penelope A. Boyden, Mark E. Anderson, Peter J. Mohler, Thomas J. Hund

**Affiliations:** 1 Department of Internal Medicine, Division of Cardiovascular Medicine, University of Iowa Carver College of Medicine, Iowa City, Iowa, United States of America; 2 Department of Molecular Physiology and Biophysics, University of Iowa Carver College of Medicine, Iowa City, Iowa, United States of America; 3 Department of Pharmacology, Center for Molecular Therapeutics, Columbia University, New York, New York, United States of America; University of California, San Diego, United States of America

## Abstract

Calmodulin kinase II (CaMKII) mediates critical signaling pathways responsible for divergent functions in the heart including calcium cycling, hypertrophy and apoptosis. Dysfunction in the CaMKII signaling pathway occurs in heart disease and is associated with increased susceptibility to life-threatening arrhythmia. Furthermore, CaMKII inhibition prevents cardiac arrhythmia and improves heart function following myocardial infarction. Recently, a novel mechanism for oxidative CaMKII activation was discovered in the heart. Here, we provide the first report of CaMKII oxidation state in a well-validated, large-animal model of heart disease. Specifically, we observe increased levels of oxidized CaMKII in the infarct border zone (BZ). These unexpected new data identify an alternative activation pathway for CaMKII in common cardiovascular disease. To study the role of oxidation-dependent CaMKII activation in creating a pro-arrhythmia substrate following myocardial infarction, we developed a new mathematical model of CaMKII activity including both oxidative and autophosphorylation activation pathways. Computer simulations using a multicellular mathematical model of the cardiac fiber demonstrate that enhanced CaMKII activity in the infarct BZ, due primarily to increased oxidation, is associated with reduced conduction velocity, increased effective refractory period, and increased susceptibility to formation of conduction block at the BZ margin, a prerequisite for reentry. Furthermore, our model predicts that CaMKII inhibition improves conduction and reduces refractoriness in the BZ, thereby reducing vulnerability to conduction block and reentry. These results identify a novel oxidation-dependent pathway for CaMKII activation in the infarct BZ that may be an effective therapeutic target for improving conduction and reducing heterogeneity in the infarcted heart.

## Author Summary

Calmodulin kinase II (CaMKII) is a multifunctional serine/threonine kinase that regulates diverse functions in heart. Recently, a novel pathway for CaMKII activation was discovered where oxidation of the kinase at specific methionine residues produces persistent activity. This alternative oxidation-dependent pathway has important implications for heart disease where oxidative stress is increased (e.g., heart failure and following myocardial infarction). We hypothesized that myocardial infarction caused by occlusion of a coronary artery would increase levels of oxidized CaMKII. Moreover, we hypothesized that oxidative CaMKII activation represents an important mechanistic link between increased oxidative stress and life-threatening heart rhythm disturbances (arrhythmias) in heart disease. We report a dramatic increase in levels of oxidized CaMKII following myocardial infarction in the canine. Based on these experimental data, we developed a novel mathematical model of CaMKII activity to study the role of oxidation-dependent CaMKII activation in regulating cardiac cell excitability. Our findings identify a novel role for oxidation-dependent CaMKII activation following myocardial infarction and provide a mechanistic link between oxidative stress and lethal cardiac arrhythmias in heart disease.

## Introduction

Calmodulin kinase II (CaMKII) mediates diverse roles in the heart, including excitation-contraction coupling, sinus node automaticity, apoptosis, hypertrophy, and gene transcription [1],[2]. Mounting experimental evidence demonstrates an important role for CaMKII in heart disease and arrhythmias. Specifically, CaMKII overexpression occurs in human heart failure [3] and transgenic mice overexpressing CaMKII develop dilated cardiomyopathy [4],[5]. Conversely, transgenic inhibition of CaMKII prevents structural remodeling and improves heart function following myocardial infarction (MI) [6] while knockout mice lacking the predominant cardiac CaMKII isoform (CaMKIIδ) are resistant to development of pressure overload-induced hypertrophy and/or heart failure [7],[8]. Finally, CaMKII inhibition prevents arrhythmias in several different mouse models of heart disease [9],[10].

CaMKII is activated by binding of Ca^2+^/calmodulin and may undergo inter-subunit autophosphorylation that allows the kinase to retain activity even upon dissociation of Ca^2+^/calmodulin (autonomy) [11]. Recently, a novel CaMKII activation pathway was identified where oxidation at specific methionine residues in the CaMKII regulatory subunit results in persistent activity independent of autophosphorylation [12]. While oxidative-dependent CaMKII activation has been shown to mediate apoptosis in response to chronic AngII treatment in the mouse [12] as well as arrhythmogenic afterdepolarizations in isolated cardiomyocytes treated with hydrogen peroxide [13], nothing is known about its role in large animal models of heart disease. Considering that levels of reactive oxygen species (ROS) such as H_2_O_2_ and superoxide are elevated following myocardial infarction [14], we hypothesized that oxidation of CaMKII represents an important pathway for CaMKII activation in the infarct border zone (BZ) that may provide a mechanistic link between increased ROS production, Na^+^ channel remodeling and conduction slowing following MI.

In this study, we describe a dramatic increase in levels of oxidized CaMKII in a well-validated large animal model of arrhythmias following MI [15]–[22]. To investigate a role for oxidized CaMKII in regulating refractoriness and conduction in the infarct BZ, we develop a novel mathematical model of CaMKII activity that includes oxidation and autophosphorylation activation pathways. Our computer simulations show that enhanced CaMKII activity in the BZ, due primarily to increased oxidation, leads to slowed conduction, prolonged refractory periods and increased vulnerability to conduction block at the BZ margin (a prerequisite for reentry initiation). Our results identify oxidation-dependent CaMKII activation as a potential link between oxidative stress and electrical remodeling after myocardial infarction. Furthermore, our findings support CaMKII inhibition as a potential therapy for reducing susceptibility to ventricular tachycardia by improving conduction and reducing refractory gradients in the infarcted heart. Finally, it is important to note the oxidative activation of CaMKII allows for independent regulation of the kinase by a host of unique upstream activators and signaling partners (e.g. oxidases/reductases) with great potential relevance to human disease. As details emerge regarding regulation of the kinase by this newly identified pathway, they may be incorporated into our model to study electrophysiological consequences of CaMKII activation via this independent signaling pathway.

## Materials and Methods

### 

#### Experimental model of myocardial infarction and immunoblotting

Myocardial infarction (MI) was produced by total coronary artery occlusion, as described previously [22]. A cardiectomy was performed five days after surgery and thin tissue slices from visible epicardial BZ and from a remote area away from the infarct (left ventricular base) were flash frozen for immunoblot analysis. Ventricular lysates were prepared for immunoblot analysis as described [21]. Equal quantities of protein were analyzed by SDS-PAGE (3–8% Tris acetate gels) under non-reducing conditions [12]. Immunoblotting was performed using a validated antiserum to oxidized CaMKII [12]. Slight differences in protein loading were corrected using an internal control standard (rabbit polyclonal antibody to actin (Santa Cruz)).

#### Animal information

This investigation used adult mongrel dogs (12 to 15 kg, 2 to 3 years old) and conforms to the Guide for the Care and Use of Laboratory Animals published by the National Institutes of Health (Pub. No. 85-23,1996).

#### Fiber model

A multicellular fiber comprised of 200 cells in a serial arrangement was used to simulate action potential (AP) propagation through normal and border zone tissue as described previously [23]. Briefly, Equation 1 describing axial current flow along the theoretical fiber was discretized and solved numerically by the Crank-Nicholson implicit method:
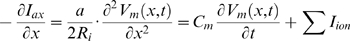
(1)where *I_ax_* is the axial current, *a* is the fiber radius (0.0011 cm), *R_i_* is the axial resistance per unit length (Ω.cm, composed of *R_myo_* (150 Ωcm) and *R_g_* (1.5 Ωcm^2^)), *C_m_* is the membrane capacitance (1 µF/cm^2^), and *I_ion_* is the transmembrane current density. A discretization element of Δx = 0.01 cm corresponding to one cell length was used in all simulations. An adaptive time step (Δt) was implemented that solves for transmembrane currents and *V_m_* along the fiber with Δt = 5 µs during AP wavefront propagation, Δt = 10 µs during repolarization, and Δt = 50 µs during diastole. Solutions using the adaptive timestep were verified to be within 1% of those using a constant Δt = 5 µs.

Transmembrane currents and ion concentration changes at each cell in the fiber are described by the Hund-Rudy dynamic (HRd) model of the canine epicardial myocyte [20],[24]. Modifications to the HRd equations to account for experimentally measured remodeling changes to several major ion channels in the infarct border zone [20] were used to represent each cell in the BZ fiber model. Equations differing from the original publications and variable definitions may be found in supplementary information (Text S1 and Table S1).

#### Pacing protocol

The fiber was paced at one end to steady state using a conservative current stimulus [25] (cycle length  = 500 ms, stimulus amplitude  = −450 µA/µF, stimulus duration  = 0.5 ms). The steady-state values for all state variables were used as initial conditions (Table S2) for subsequent simulations.

#### Statistics

When appropriate, differences between groups were analyzed with ANOVA and least squares difference post-hoc test. A value of p<0.05 was considered statistically significant. Values are expressed as mean ± SD.

## Results

### CaMKII is oxidized in the infarct border zone

**Figure 1:**
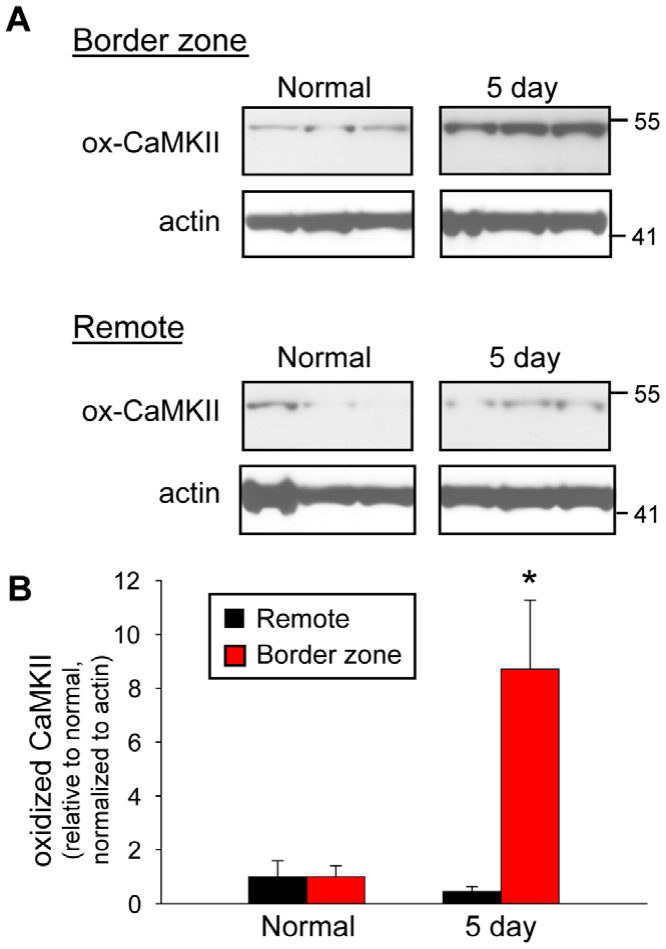
Increased levels of oxidized CaMKII following myocardial infarction. (A) Representative immunoblots and (B) densitometric measurements (normalized to actin and expressed relative to normal levels) of oxidized CaMKII from remote and BZ regions of normal and infarcted hearts. Error bars designate standard deviation (*p<0.01 compared to normal BZ or remote, n = 4).

Based on the recent discovery of a novel oxidation-dependent pathway for CaMKII activation [12], immunoblot analysis was first performed in a well-validated large animal model of arrhythmias [15]–[22] to determine whether oxidization of CaMKII occurs in the infarct BZ five days post-occlusion (Figure 1). Interestingly, levels of oxidized CaMKII were over eight-fold greater in the five-day BZ compared to normal (non-infarcted) (p<0.01), but were unchanged in remote regions of the same hearts (Figure 1, p = NS vs. normal). These data together with our previous findings that CaMKII autophosphorylation is significantly increased in the five-day infarct BZ [20] indicate that CaMKII activity is enhanced in the infarct BZ.

### Model of oxidative CaMKII activation and action potential propagation

**Figure 2:**
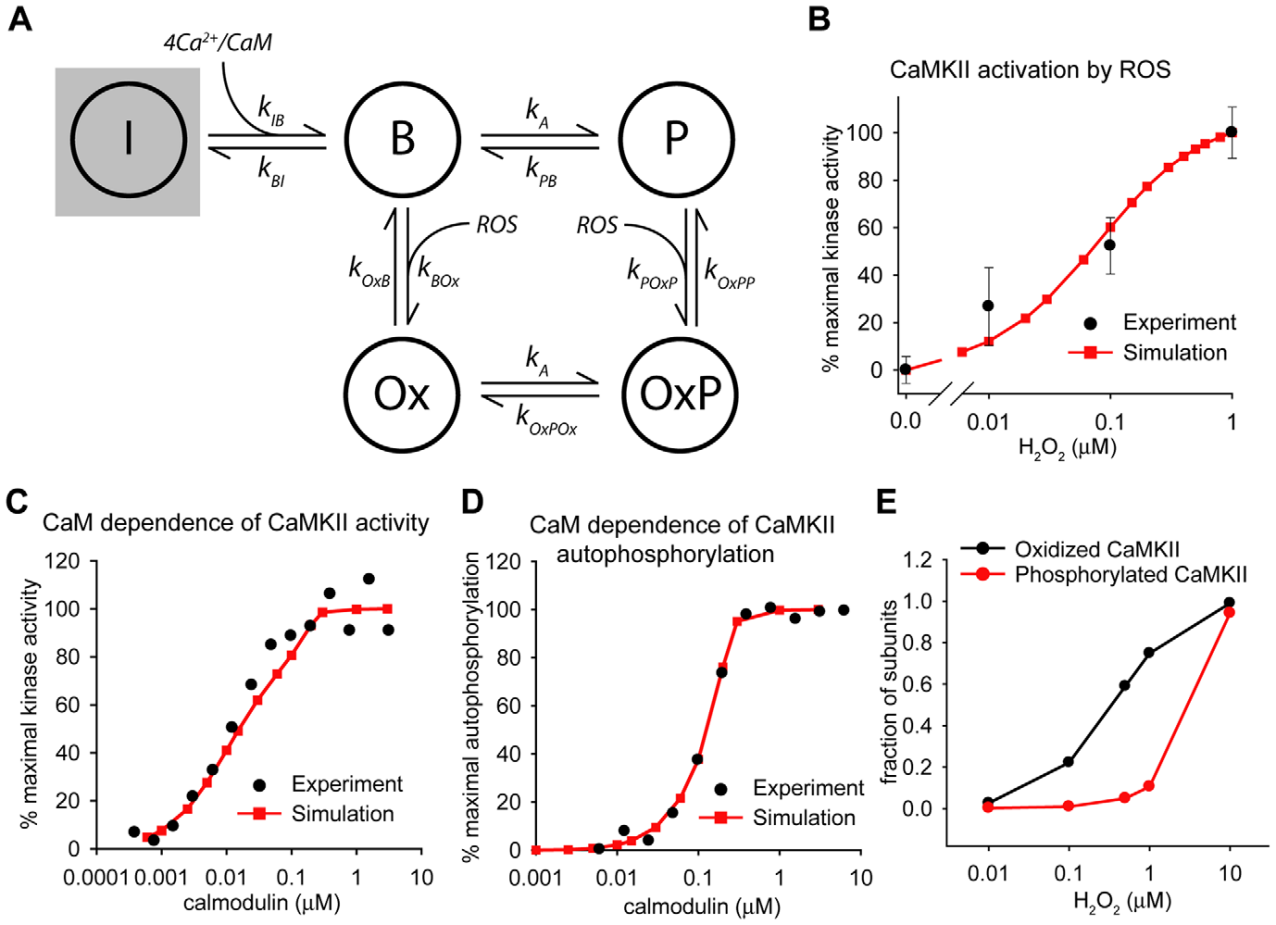
Mathematical model of CaMKII activity. (A) State diagram for CaMKII including activation by Ca^2+^/CaM (*B*), oxidation (*Ox*), and autophosphorylation (*P*). Rate constants are provided in supplementary information (Text S1 and Table S3). (B) Simulated dose-dependent activation of CaMKII by H_2_O_2_ compared to experiment [12]. Ca^2+^/CaM  = 1 µM and autophosphorylation rate  = 0 in simulations corresponding to the following experimental conditions: 200 µM Ca^2+^, 1 µM CaM in the absence of ATP (to prevent autophosphorylation). Simulated CaM dependence of (C) CaMKII activity and (D) autophosphorylation compared to experiment [50]. Saturating conditions for Ca^2+^ (Ca^2+^ = 0.5 mM) are used in experiment and simulation to allow for control of [Ca^2+^/CaM] by varying [CaM]. (E) Simulated levels of CaMKII oxidation and autophosphorylation in the BZ model for different levels of oxidative stress (model paced to steady-state at cycle length of 500 ms).

To determine whether enhanced CaMKII activity, due in part to oxidation (Figure 1), regulates conduction in the infarct BZ, we revised our model of the canine ventricular action potential [20],[24] to include a new model of CaMKII activity based on the simplified scheme proposed by Dupont et al [26]–[28] (Figure 2). Importantly, our model includes an oxidized active state in addition to a Ca^2+^/CaM bound active state and an autophosphorylated active state. Inclusion of an additional autonomous active state (Ca^2+^/CaM dissociates from phosphorylated subunit) was found to have no impact on model behavior (state occupancy <0.001%, not shown) and was therefore not included in the final model. Consistent with experimental observations [12], Ca^2+^/CaM must bind to a subunit before oxidation may occur (no direct transition from inactive to oxidized active state). Rate constants for state transitions were taken from the literature or chosen to fit experimental data (Table S3, Figure 2B–D). Model equations are provided in supplementary information (Text S1). Our experimental data demonstrate a significant increase in both oxidized (Figure 1) and autophosphorylated CaMKII [20] in the infarct BZ. Even though autophosphorylation and oxidation occur through distinct pathways, the model assumes that the same subunit may be both oxidized and autophosphorylated. Furthermore, consistent with previous work [26]–[28], the model assumes that any active subunit (including oxidized) may autophosphorylate another Ca^2+^/CaM bound subunit. Thus, the model predicts a secondary increase in the fraction of autophosphorylated CaMKII subunits with an increase in oxidized subunits due to oxidative stress (Figure 2E). Currently, the upstream pathways responsible for increased CaMKII autophosphorylation are unknown. However, our model predicts that oxidative stress may account for the increase in both oxidized and autophosphorylated subunits measured in the infarct BZ (Figure 2E). Thus, for the purpose of this study, we assume that the primary defect responsible for activated CaMKII in the BZ is oxidative stress.

While absolute measures of H_2_O_2_ levels are limited (likely less than 0.25 µM at baseline [29],[30]), an increase in ROS levels from 10- to 100-fold have been reported following ischemia-reperfusion [31]–[33]. Furthermore, ROS levels of 10 µM *in vitro* have been shown to recapitulate the level of oxidative stress observed *in vivo* in the BZ [34]. Unless otherwise stated, we assume [ROS]  = 1.0 µM in the BZ, likely a conservative estimate. However, based on the fact that the exact level of ROS is unknown in the BZ and is likely to be highly heterogeneous, we also explore a range of ROS levels from 0 to 10 µM. Note that for [ROS]  = 1 µM, the fraction of autophosphorylated subunits in the BZ is much lower than the fraction of oxidized subunits (0.11 for autophosphorylation compared to 0.75 for oxidation, Figure 2E), indicating that oxidation rather than autophosphorylation is the primary determinant of increased CaMKII activity in the BZ model.

**Figure 3:**
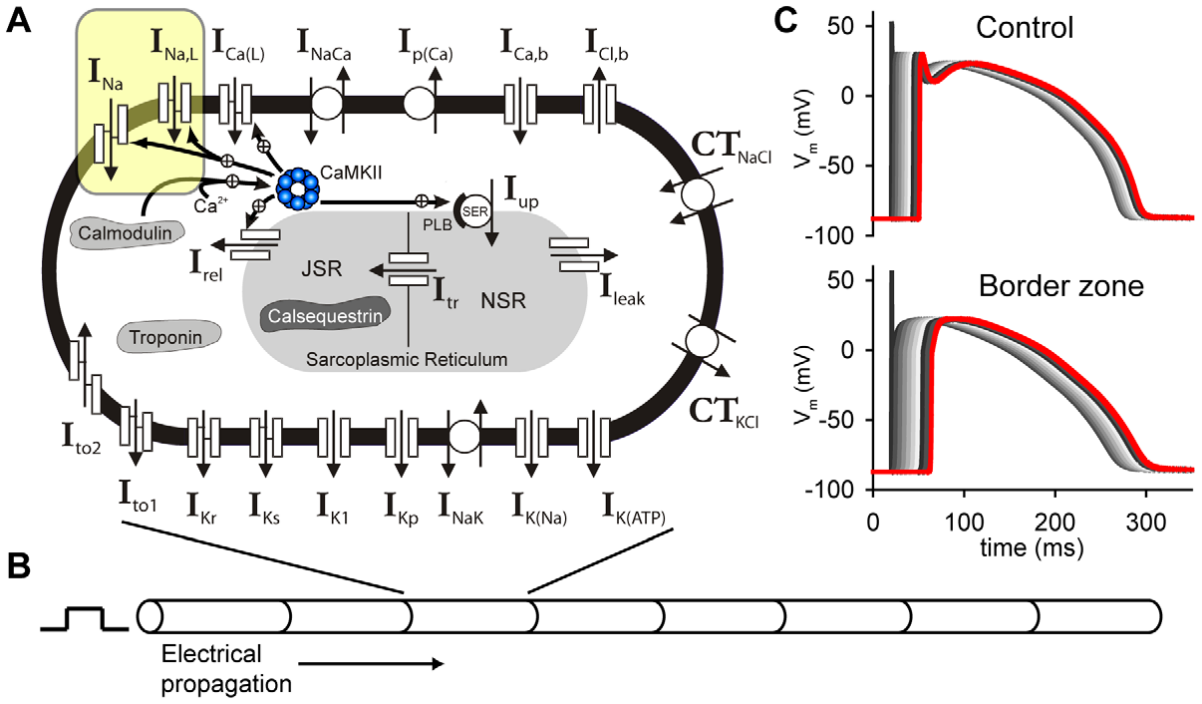
Mathematical model of cardiac action potential and electrical conduction. (A) Hund-Rudy dynamic (HRd) canine ventricular epicardial cell model. Symbols are defined in text and in Table S1. (B) One-dimensional fiber model comprised of individual cells electrically coupled through gap junctions. A current stimulus is applied at the end of the fiber (cell 1) and the excitation wavefront propagates down the fiber. (C) Simulated action potentials from every 20^th^ cell in the control (*top*) and border zone (*bottom*) fibers.

Importantly, our model of the BZ myocyte also accounts for observed remodeling changes to the density and/or kinetics of several ion channels, including the L-type Ca^2+^ current, transient outward K^+^ current, and Na^+^ current [20]. Specifically for the Na^+^ current, changes to kinetics and peak current have been observed [22]. Since CaMKII has been shown to alter Na^+^ channel kinetics but not peak current, our model assumes that the reduction in total Na^+^ channel density occurs through a CaMKII-independent pathway [20]. NZ (control) and BZ cell models are incorporated into one-dimensional fibers to study conduction (Figure 3).

### CaMKII regulates *I_Na_* inactivation in border zone

**Figure 4:**
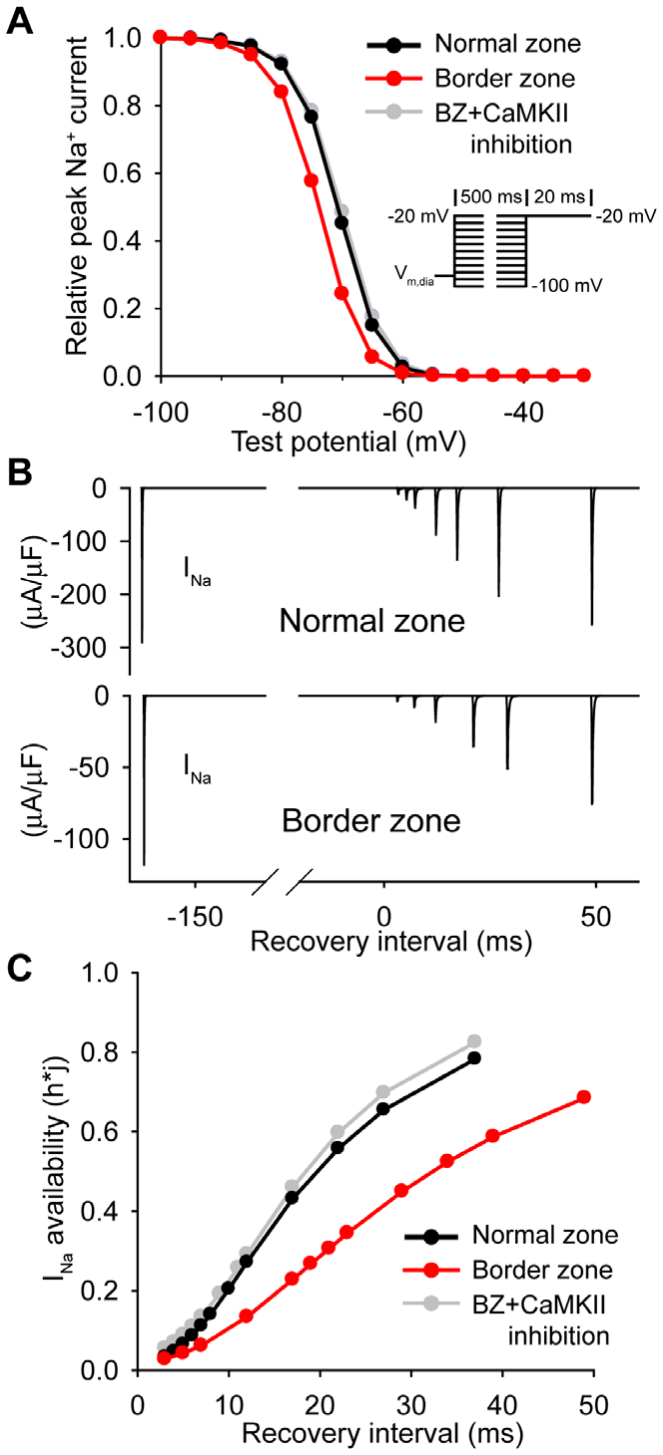
CaMKII regulates *I_Na_* in the infarct border zone. (A) Steady-state *I_Na_* inactivation curves in NZ and BZ models (pulse protocol shown in *inset*). Inhibition of the CaMKII pathway (CaMKII activity held constant at zero) eliminates differences between NZ and BZ *I_Na_* steady-state inactivation. (B) Simulated Na^+^ currents in control (*top*) and border zone (*bottom*) models during application of a premature stimulus to cell 1 at varying S1S2 interval to determine recovery from inactivation. (C) *I_Na_* availability (h*j) in control and border zone models with and without CaMKII inhibition. *I_Na_* recovery is dramatically slower in the border zone fiber compared to control. CaMKII inhibition in the BZ accelerates recovery from inactivation to a rate similar to NZ (recovery curve for NZ model + CaMKII inhibition superimposes curve for BZ model + CaMKII inhibition, not shown).

*I*_Na_ inactivation and recovery from inactivation were first determined in the NZ and BZ fiber after pacing to steady state (Figure 4). *I*_Na_ recovery from inactivation was determined by applying a premature stimulus (S2) at a varying S1S2 interval and plotting channel availability (calculated as product of inactivation gates, h*j) vs. recovery interval (S1S2 interval - APD_90_). *I*_Na_ steady-state inactivation is shifted to more hyperpolarized potentials (Figure 4A) and recovery from inactivation is slower (Figure 4C) in the BZ fiber compared to NZ, consistent with our single cell simulations [20] and experimental measurements [22]. CaMKII inhibition (CaMKII activity held constant at zero) shifts *I_Na_* steady state inactivation to more depolarized potentials (Figure 4A) and accelerates recovery from inactivation in the BZ fiber (Figure 4C) but has little effect in NZ (steady-state inactivation and recovery curves superimpose curves from BZ+CaMKII inhibition model, not shown). Thus differences in *I*_Na_ inactivation between NZ and BZ observed under control conditions are largely eliminated upon CaMKII inhibition (Figure 4).

### CaMKII regulates conduction in border zone

**Figure 5:**
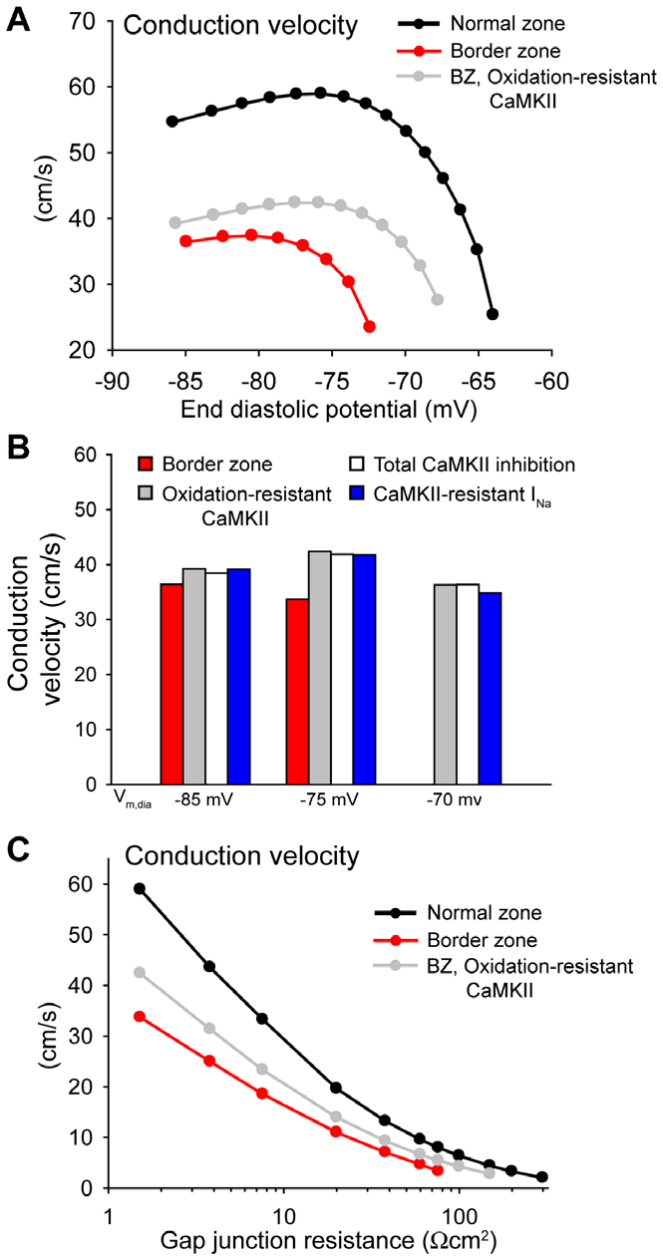
CaMKII regulates conduction in the infarct border zone. (A) Conduction velocity vs. end diastolic potential (V_m,dia_) in normal and border zone fibers. [K^+^]_o_ is increased incrementally from 5.4 mM to 13 mM to depolarize V_m,dia_ from −87 to −63 mV. Elimination of oxidation-dependent CaMKII activation (oxidation-resistant CaMKII) increases conduction velocity at all V_m,dia_ and extends the range of V_m,dia_ over which successful conduction occurs in the border zone fiber. (B) Total CaMKII inhibition and eliminating CaMKII-dependent effects on *I_Na_* have a similar effect on conduction velocity as making CaMKII resistant to oxidation. (C) Conduction velocity vs. gap junction resistance (R_g_) in NZ and BZ fibers ([K^+^]_o_  = 8.0 mM). Eliminating oxidation-dependent CaMKII activation improves conduction in the BZ fiber even at very high degrees of cell uncoupling (R_g_ up to 150 Ωcm^2^).

Based on the effects of CaMKII on *I*_Na_ availability, we hypothesized that enhanced CaMKII activity would promote slow conduction in the BZ. While resting transmembrane potential is comparable between isolated BZ and NZ myocytes [19], membrane depolarization is observed in multicellular BZ preparations [35],[36]. Therefore, we measured conduction velocity in NZ and BZ fibers over a range of end diastolic potentials (V_m,dia_, −86 to −63 mV), by increasing [K^+^]_o_ incrementally from 5.4 to 13 mM. Conduction velocity was measured across the central 100 cells (Figure 5). Conduction was dramatically slower at every V_m,dia_ in the BZ compared to NZ (65–100% slower) (Figure 5A). Furthermore, while successful conduction was observed in the NZ for potentials up to −64 mV, conduction block occurred in the BZ for V_m,dia_ >−72 mV. In fact, conduction velocity is steeply dependent on the concentration of ROS in the BZ over a range of concentrations from about 0.01 µM to 1 µM (Figure S1). To determine the role of oxidation-dependent CaMKII activity in conduction slowing in the BZ, we measured conduction velocity in the BZ model resistant to CaMKII oxidation (CaMKII_ox_  = 0). Making CaMKII resistant to oxidation increased conduction velocity at all V_m,dia_ in the BZ with a greater effect at more depolarized V_m,dia_ (Figure 5A). Moreover, successful conduction was restored in the BZ for V_m,dia_ up to −68 mV (compared to −64 mV in the control). In contrast, the BZ model resistant to CaMKII autophosphorylation showed very little improvement in conduction (Figure S2). Furthermore, inhibiting total CaMKII activity showed a similar improvement in conduction as the oxidation-resistant model (Figure 5B), indicating that oxidation is the primary determinant of enhanced kinase activity in our BZ model. To verify that CaMKII-dependent effects on conduction were mediated by regulation of *I*_Na_ kinetics, we also calculated conduction velocity in the BZ model with *I*_Na_ resistant to CaMKII phosphorylation. As expected, this model showed a similar improvement in conduction as the oxidation-resistant model (Figure 5B), indicating that enhanced CaMKII activity regulates conduction by altering *I*_Na_ kinetics. In summary, enhanced CaMKII activity contributes to reduced conduction velocity in the BZ fiber, even promoting conduction block in the setting of depolarized transmembrane potential.

Remodeling in BZ tissue involves not only changes to ion channel properties and rest potential, but also intracellular communication [37],[38]. In order to address whether cellular uncoupling affects the role of oxidized CaMKII in regulating conduction, we determined conduction velocity in the fiber over a range of gap junction resistances (Figure 5C). Increasing gap junctional resistance (R_g_) from 1.5 to 60 Ωcm^2^ produced a similar decrease in conduction velocity in NZ and BZ fibers (86% and 90% decrease, respectively). Successful conduction occurred in the NZ fiber for R_g_ up to 300 Ωcm^2^, while conduction block was observed in the BZ fiber for R_g_>76 Ωcm^2^. Eliminating oxidation-dependent CaMKII activity increased conduction velocity in the BZ fiber at all R_g_ and restored conduction for R_g_ up to 150 Ωcm^2^ (Figure 5C), indicating that CaMKII regulates conduction even in the setting of gap junction uncoupling.

### CaMKII regulates effective refractory period in border zone

**Figure 6:**
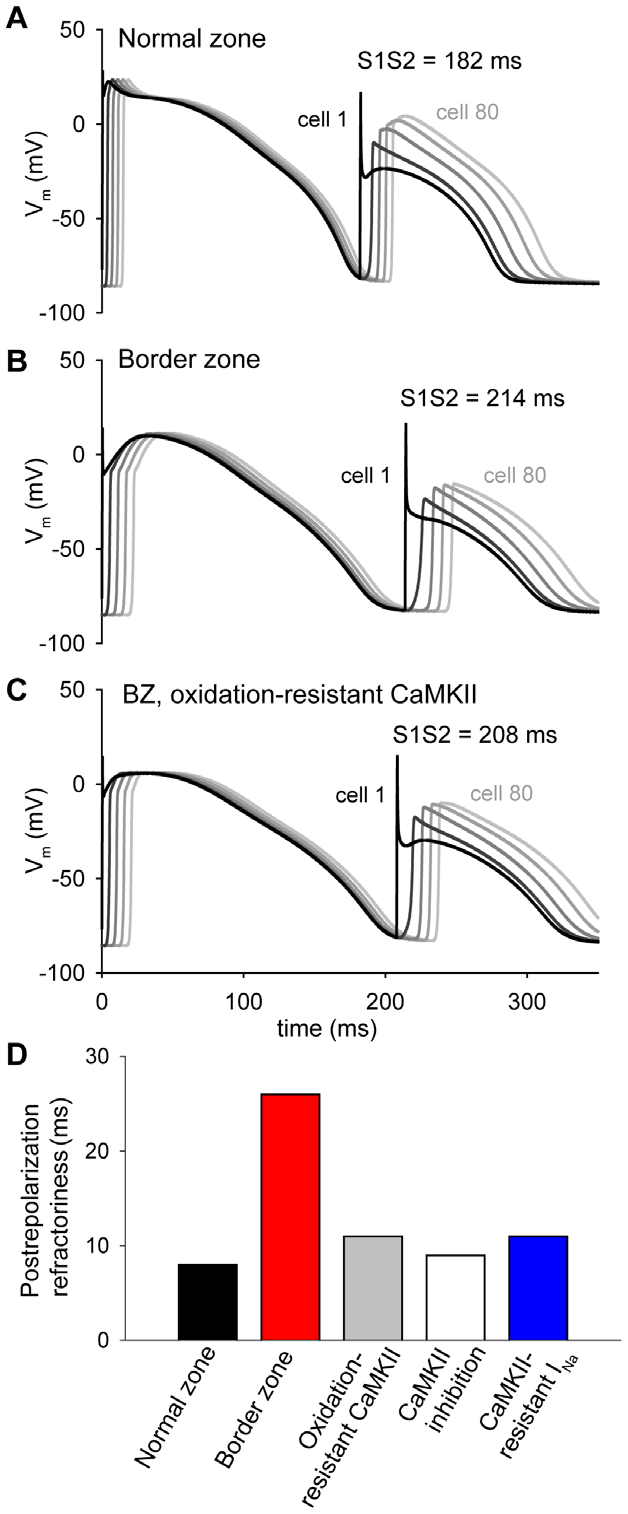
CaMKII regulates effective refractory period in the infarct border zone. A premature stimulus is applied to cell 1 at varying S1S2 intervals to determine the effective refractory period in (A) NZ, (B) BZ, and (C) BZ with oxidation-resistant CaMKII. Simulated action potentials are shown from cells 1, 20, 40, 60, and 80 along the fiber for the shortest S1S2 interval that generates a successfully propagating action potential. While ERP is longer in the BZ than in NZ (213 ms and 181 ms, respectively), elimination of oxidation-dependent CaMKII activation reduces these differences. (D) Postrepolarization refractoriness (ERP-APD_90_) in NZ, BZ, BZ with oxidation-resistant CaMKII, BZ with total CaMKII inhibition, and BZ with CaMKII-resistant *I_Na_*.

Effective refractory period (ERP) of the action potential is dramatically prolonged in BZ compared to NZ, despite comparable action potential durations [18],[19],[35]. Moreover, large gradients in refractoriness at the BZ margin have been associated with conduction block and the initiation of reentrant arrhythmias [15],[17],[18]. Based on these data and the ability of CaMKII to regulate *I*_Na_ recovery from inactivation (Figure 4C), we hypothesized that enhanced CaMKII activity would contribute to prolonged refractoriness in the BZ. To test our hypothesis, ERP was determined in NZ and BZ fibers by applying a premature (S2) stimulus during the repolarization phase of the action potential at cell 1 (site of S1 stimulus). The S1S2 interval was increased until a second propagating wave was generated in the wake of the final S1 stimulated AP (Figure 6). ERP is defined as the largest S1S2 interval that fails to generate a propagating excitation wave and is a function of both action potential duration (APD) and postrepolarization refractoriness. Consistent with experiment [18],[19],[35], ERP is much greater in the BZ model (213 ms compared to 181 in the NZ) (Figure 6). Small differences in APD (173 ms and 187 ms in NZ and BZ, respectively) account for only a portion of this difference in ERP. Rather the primary determinant of prolonged ERP in the BZ is increased postrepolarization refractoriness due to the much slower time course of recovery from inactivation of *I*_Na_ (Figure 4C). Making CaMKII resistant to oxidative activation reduces ERP to 207 ms in the BZ model despite a slight prolongation of APD (Figure 6C) by eliminating differences in postrepolarization refractoriness (measured as ERP – APD, Figure 6D). Likewise, total CaMKII inhibition and making *I*_Na_ resistant to CaMKII phosphorylation reduce ERP by normalizing postrepolarization refractoriness (Figure 6D). These results suggest that oxidation-dependent CaMKII activation contributes to large gradients of refractoriness, particularly at the margins of the infarct BZ, by regulating *I*_N*a*_ kinetics.

### CaMKII increases vulnerability to conduction block

**Figure 7:**
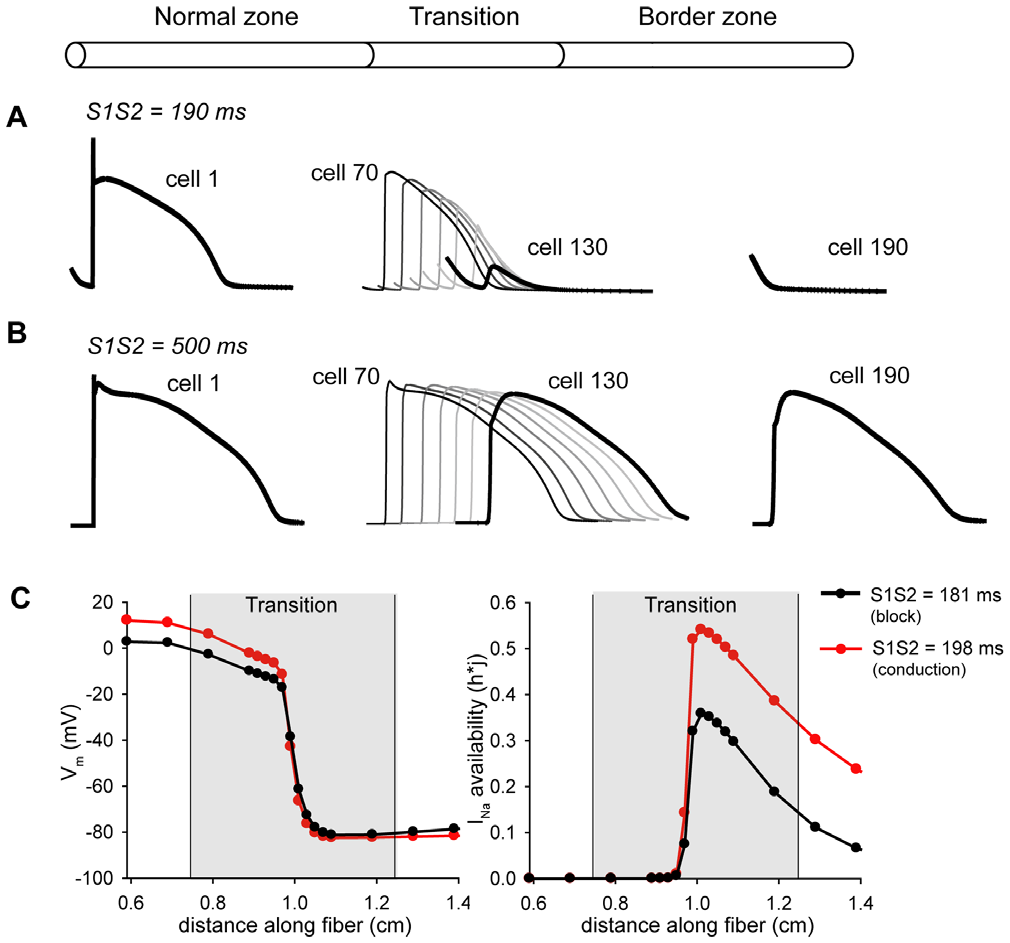
Role of CaMKII in formation of conduction block at the border zone margin. (A) Conduction block in a one-dimensional fiber comprised of NZ (cells 1–75), BZ (cells 126–200), and transitional (cells 76–125) regions. A premature S2 stimulus is applied at cell 1 to induce conduction block at the BZ margin (S1S2  = 190 ms). (B) Application of S2 at a coupling interval greater the vulnerable window (S1S2  = 500 ms) results in successful conduction through the entire fiber. (C) V_m_ and *I_Na_* availability (h*j) along the fiber as the wavefront reaches the BZ margin for a premature stimulus that blocks (S1S2  = 181 ms, black lines) or propagates successfully through the entire fiber (S1S2  = 198 ms, *red lines*).

Electrophysiological mapping during programmed stimulation to induce ventricular tachycardia has revealed that premature excitation block occurs in areas of large refractory gradients at the BZ margin [18]. Our findings that enhanced CaMKII activity substantially increases ERP in the BZ led us to hypothesize that CaMKII promotes formation of conduction block at the transition between normal and border zone tissue by introducing large refractory gradients. To test our hypothesis, we used a heterogeneous fiber comprised of coupled NZ (cells 1–75) and BZ (cells 126–200) cells with a central transitional region (cells 76–125) across which BZ parameters were linearly scaled. The size of the transitional region corresponds to the approximate width of the outer common pathway (about 5.0 mm [15]) (Figure 7A). The fiber was paced to steady state at cell 1 and a premature S2 stimulus was applied at the same cell. The S1S2 pacing interval was varied to determine the critical range of S1S2 intervals (vulnerable window, VW) that resulted in conduction block at the transition from the NZ into the BZ region. S1S2 intervals from 181 to 197 ms (VW = 18 ms) resulted in an action potential that propagated successfully through the NZ region but failed at the transition into the BZ region (Figure 7A). S1S2 pacing intervals shorter than this critical range failed to propagate even through the NZ region while S1S2 intervals greater than this range successfully propagated through the entire fiber (Figure 7B). V_m_ and *I*_Na_ availability (h*j) spatial profiles as the wavefront reaches the BZ margin indicate that *I*_Na_ availability is a critical determinant of whether or not a premature wavefront blocks at the BZ margin (Figure 7C).

**Figure 8:**
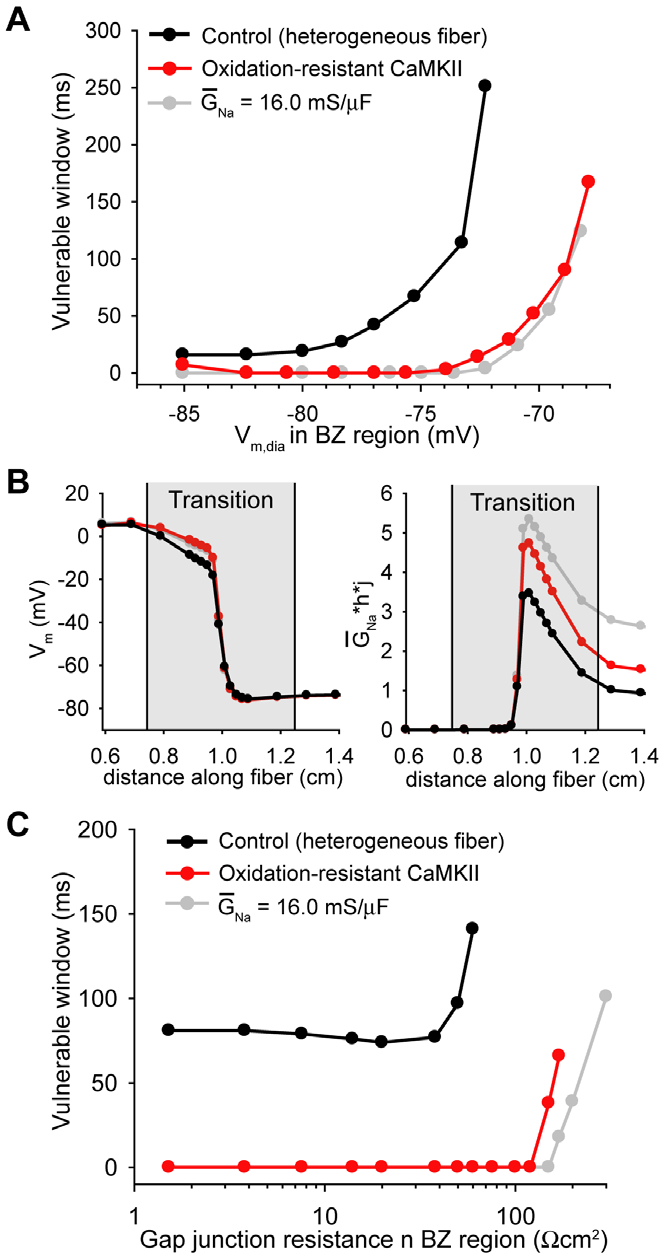
Vulnerable window for conduction block. (A) Range of S1S2 intervals that result in conduction block at the BZ margin (vulnerable window) as a function of end diastolic potential (V_m,dia_) in the BZ region. Results are shown for the fiber with heterogeneous properties (control, *black line*), with oxidation-resistant CaMKII (*red line*), and with normal *I_Na_* conductance throughout (*gray line*). Eliminating oxidation-dependent CaMKII activity or heterogeneity in *I_Na_* conductance greatly reduces the VW at all V_m,dia_. (B) V_m_ and effective *I_Na_* availability (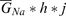) along the fiber as the action potential wavefront reaches the BZ margin for the fiber with heterogeneous properties (control, *black lines*), with oxidation-resistant CaMKII (*red lines*), and with normal *I_Na_* conductance throughout (*gray lines*). [K^+^]_o_ = 8.0 mM in the BZ region (and scaled linearly across the transition region). (C) Vulnerable window as a function of gap junction resistance (R_g_) in the BZ region for the fiber with heterogeneous properties (control, *black line*), with oxidation-resistant CaMKII (*red line*), and with normal *I_Na_* conductance throughout (*gray line*). Eliminating oxidation-dependent CaMKII activity or normalizing *I_Na_* conductance reduces VW at all R_g_.

Figure 8 shows VW in the heterogeneous fiber as a function of V_m,dia_ in the BZ region ([K^+^]_o_ was scaled linearly across the transition region as with other BZ parameters). VW shows a monophasic increase as V_m,dia_ is depolarized from −85 mV to −72 mV. V_m,dia_ greater than −72 mV results in transient block at the BZ margin even at the basic cycle length of 500 ms. Making CaMKII resistant to oxidative activation greatly reduces VW at all V_m,dia_ (Figure 8A). Furthermore, transient block is not observed at the basic cycle length until V_m,dia_ is depolarized above −68 mV. Total CaMKII inhibition and making *I*_Na_ resistant to CaMKII phosphorylation also effectively prevented formation of block at the BZ margin (VW less than 1 ms for V_m,dia_ up to −72 mV and −74 mV, respectively, not shown).

While these data show that oxidation-dependent CaMKII activation increases the vulnerability to conduction block at the BZ margin, a prerequisite for initiation of reentrant arrhythmias, it is important to note that CaMKII-independent remodeling of ion channels (notably *I*_Na_) also likely play an important role. To address the role of CaMKII-independent remodeling in conduction block, we measured VW in the heterogeneous fiber with normal *I*_Na_ conductance throughout. Eliminating differences in *I*_Na_ conductance successfully reduced vulnerability to transient conduction block across a wide range of V_m,dia_ (Figure 8A), indicating that both CaMKII-dependent (altered kinetics) and CaMKII-independent (reduced channel conductance) effects on *I*_Na_ increase the vulnerability to conduction block and reentrant arrhythmias at the BZ margin. Interestingly, eliminating either CaMKII-dependent (oxidation-resistant CaMKII) or CaMKII-independent (normal 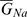) heterogeneities in the fiber resulted in a similar increase in the effective *I*_Na_ availability (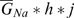) in the transition region (Figure 8B).

We next determined whether cellular uncoupling in the BZ region would alter the role of CaMKII-dependent or CaMKII-independent *I*_Na_ changes in formation of conduction block at the BZ margin (Figure 8C). Increasing R_g_ in the BZ region (R_g_ scaled linearly across the transition region as with other BZ parameters) had very little effect on VW for a moderate degree of uncoupling (R_g_ up to 38 Ωcm^2^). However, VW increased sharply for R_g_>38 Ωcm^2^ until block occurred at the BZ margin even at the basic cycle length (R_g_>60 Ωcm^2^). Eliminating oxidation-dependent CaMKII activation or normalizing *I*_Na_ conductance reduced VW at all R_g_ (VW = 0 ms for R_g_ up to 120 Ωcm^2^ and 150 Ωcm^2^, respectively) indicating that CaMKII-dependent and CaMKII-independent effects on *I*_Na_ regulate VW even in the presence of cellular uncoupling.

## Discussion

Our data provide the first evidence for oxidation of CaMKII as an important component of the remodeling process following MI. Furthermore, our simulation results provide the following insight into regulation of CaMKII signaling by this novel oxidative pathway: 1) Significant oxidative activation of the kianse occurs under pathophysiological conditions; 2) Oxidative stress may activate the kinase not only through direct oxidation but also through a secondary increase in autophosphorylation; and 3) Changes in Na^+^ channel kinetics due to oxidative CaMKII activation are sufficient to impact conduction in the BZ.

Conduction in the canine infarct border zone is highly irregular with areas of very slow and discontinuous conduction during sinus rhythm [17]. During programmed stimulation, lines of conduction block often form transverse to the fiber axis leading to initiation of reentry and sustained ventricular tachycardia [15],[16]. The mechanisms responsible for conduction block and reentry in the BZ are unknown although remodeling changes in tissue refractoriness and electrical coupling most likely play important roles [15]–[18]. At the cellular level, it is clear that Na^+^ channel dysfunction contributes to reduced action potential upstroke and action potential amplitude in myocytes isolated from the infarct border zone [19],[22]. Previous modeling studies have shown that decreased Na^+^ channel availability contributes to prolonged refractoriness in BZ cells despite AP duration comparable to NZ [39] and that slowing of Na^+^ channel recovery from inactivation or reducing Na^+^ channel conductance increases the vulnerable period for unidirectional block in cardiac tissue [40]. Our simulations demonstrate that CaMKII may regulate both conduction velocity and refractoriness in the BZ through its effects on voltage-gated Na^+^ channel kinetics. Furthermore, by introducing gradients in *I*_Na_ availability and refractoriness, CaMKII activation, due in part to oxidation, increases vulnerability to conduction block at the BZ margin, a prerequisite for reentrant excitation. Moreover, our simulations suggest that CaMKII inhibition improves conduction (particularly in depolarized tissue) and reduces ERP in the BZ, thereby reducing the risk for conduction block and reentrant excitation. These results are significant in light of experimental mapping studies showing premature excitation block in areas of large gradients in refractoriness [18]. While the current study focuses on conduction defects in the BZ, it is important to note that CaMKII activation is also expected to regulate intracellular Ca^2+^ cycling that itself may promote arrhythmias [2].

Our findings regarding the effects of CaMKII on conduction are consistent with experimental studies in mice that over-express CaMKIIδ. Specifically, consistent with our simulations, CaMKIIδ over-expression results in prolonged QRS intervals (marker of slowed intraventricular conduction) and increased arrhythmia susceptibility [41]. In contrast, our finding that CaMKII acts to increase post-repolarization refractoriness and therefore ERP in the BZ does not agree with shorter refractory periods in CaMKIIδ mice [41]. While the nature of this discrepancy is unclear, it is difficult to reconcile the reported effects of CaMKII on Na^+^ channel recovery (slowing) with the measured effects on refractoriness in transgenic mice. It is possible that the decrease in ERP measured in transgenic mice is due to secondary effects of chronic CaMKII over-expression rather than acute signaling effects. Regardless, further studies are needed to define the role of CaMKII in regulating refractoriness in the heart.

Clearly, many factors besides remodeling of voltage-gated Na^+^ channels influence conduction in the infarct BZ. Specifically, alterations in cell-to-cell coupling due to gap junction remodeling and/or fibrosis undoubtedly play an important role in abnormal conduction. In fact, studies have shown a close correlation between location of the central common pathway of the reentrant circuit and connexin43 redistribution suggesting that gap junction remodeling is required for maintenance of reentrant excitation [37],[38]. Furthermore, preferential uncoupling along the transverse fiber axis is thought to increase the degree of anisotropy in the BZ and facilitate initiation and maintenance of reentry. In addition, remodeling of the extracellular space in the BZ likely interacts with changes in gap junction coupling to affect conduction [42]. Thus, cell communication is regulated by a complex set of ion channel and structural changes in the BZ. Importantly, we find that CaMKII-dependent changes in *I*_Na_ kinetics regulate conduction in the BZ independent of the degree of cell coupling. Moreover, we report that CaMKII inhibition may restore conduction in the BZ even in the setting of very poor coupling (R_g_ up to 150 Ωcm^2^).

Previous studies have shown increased levels of ROS in five-day infarct BZ regions [34]. Moreover, exposure of cardiac Na^+^ channels expressed in HEK cells to ROS recapitulates the remodeling phenotypes observed in BZ myocytes. Our results suggest that the newly discovered oxidation-dependent pathway for CaMKII activation serves as a critical link between oxidative stress, enhanced CaMKII activity, Na^+^ channel dysfunction, and abnormal conduction in the infarct BZ. Of course, CaMKII is unlikely to be the only pathway through which oxidative stress alters cell excitability as oxidation affects many proteins in the heart, including kinases, transcription factors, ion channels, pumps, transporters, Ca^2+^ handling proteins, and contractile machinery [43].

While studies from our group and others demonstrate an important role for activated CaMKII in remodeling following MI, the upstream signaling pathways responsible for enhanced CaMKII activity remain to be fully elucidated (Figure 8). In this study, we assume that oxidative stress is the primary cause of enhanced CaMKII activity through direct oxidation of the kinase that also produces a secondary increase in the fraction of autophosphorylated subunits (Figure 2E). Clearly, CaMKII oxidation downstream of increased ROS production is one possible pathway for CaMKII activation in the BZ (Figure 1). However, a number of other upstream factors likely play an important role in regulating CaMKII activity following MI. For example, β-adrenergic stimulation, observed in the setting of myocardial infarction, activates CaMKII [6] and may also contribute to electrical remodeling after MI. Another possible mechanism for dysfunction in the CaMKII signaling pathway involves loss of coordinate regulation by phosphatases. Recently, it was discovered that miR-1 over-expression causes CaMKII-dependent hyperphosphorylation of RyR2 and afterdepolarizations due to reduced expression of the B56α regulatory subunit of the serine/threonine protein phosphatase 2A, PP2A [44]. Previous studies from our group have shown that B56α binds to, and is targeted by the adapter protein ankyrin-B in heart [45],[46]. Furthermore, B56α expression is reduced in cardiomyocytes lacking ankyrin-B [45]. More recently, we have shown that expression levels of ankyrin-B are significantly reduced in the BZ leading to altered expression and distribution of ankyrin-B associated membrane proteins including PP2A [21]. Interestingly, previous modeling studies have shown that loss of local phosphatase signaling may greatly potentiate levels of autophosphorylated CaMKII [27]. Therefore, loss of ankyrin-B may provide another mechanism for abnormal CaMKII signaling in the BZ through abnormal localization and/or activity of PP2A. Interestingly, patients with ankyrin mutations show catecholaminergic-induced afterdepolarizations [47]–[49] as observed in cells with reduced B56α. Future studies are needed to define the upstream signaling pathway(s) responsible for CaMKII activation, Na^+^ channel remodeling and increased susceptibility to reentrant arrhythmias after MI.

Finally, it is important to note that activation of CaMKII through direct oxidation has only recently been discovered. Consequently, much remains unknown regarding the signaling mechanisms that regulate this pathway. Analogous to regulation of autophosphorylation by local kinase/phosphatase activity and local concentrations of Ca^2+^/calmodulin, oxidative activation is likely controlled by a delicate balance of oxidase/reductase activity, mitochondrial function and local calcium signaling. Furthermore, degree of crosstalk between oxidative and autophosphorylation pathways and their relative importance in response to the complex set of upstream stressors in heart disease remain to be determined. These details, as they emerge, may be incorporated into our model to analyze the functional consequences of upstream signals that converge through distinct pathways to alter CaMKII activity.

### Limitations

While our mathematical model accounts for central aspects of the newly identified oxidation-dependent pathway for CaMKII activation, it has important limitations based on the available experimental data. Many questions remain to be answered regarding the function of CaMKII oxidation in the normal and diseased heart. For example, how do oxidation and autophosphorylation of CaMKII interact to control regulation of the holoenzyme? Do these pathways interact synergistically to activate the kinase and what are the unique/shared targets for each activation pathway? Finally, what is the relative importance of oxidized versus autophosphorylated CaMKII in normal and diseased hearts? Answers to these questions will not only facilitate the development of more comprehensive models but also will provide critical information necessary to design novel cell-specific therapies for regulating cardiac excitability.

It is important to note that CaMKII in the model detects a subspace pool of Ca^2+^ that reaches concentrations somewhere between cytosolic and dyadic concentrations (peak concentration 10–20 µM). Previous modeling studies have shown that the dynamic response of CaMKII may vary greatly between dyadic and cytosolic pools based on variability in concentrations of Ca^2+^ and CaM [27]. Consistent with previous studies [27], we found that cytosolic Ca^2+^ transients do not significantly activate CaMKII activity at baseline or even in the presence of 1 µM H_2_O_2_ (<1% maximal activity, not shown). However 10 µM H_2_O_2_ was able to activate CaMKII (25% maximal), suggesting that a sufficiently high level of oxidative stress may be able to activate even cytosolic CaMKII. Clearly, local regulation of CaMKII in well-defined subcellular domains is an exciting area for future research with important implications for human disease. As we learn more about CaM and CaMKII signaling in the vicinity of Na^+^ channels, it will be important to incorporate these data into the model.

## Supporting Information

Figure S1 Cell excitability as a function of ROS levels in the BZ. (A) Conduction velocity and (B) refractoriness in the infarct BZ as a function of ROS concentration. Conduction velocity is determined across the middle 100 cells in the BZ fiber. Postrepolarization refractoriness is calculated as the difference between effective refractory period and action potential duration at 90% repolarization.
(2.17 MB TIF)

Figure S2 Role of autophosphorylation-dependent CaMKII activation in regulating conduction in the infarct BZ. Conduction velocity is determined across the middle 100 cells in the BZ fiber over a range of end diastolic membrane potentials (V_m,dia_). [K^+^]_o_ is increased incrementally from 5.4 mM to 13 mM to V_m,dia_ from −87 to −63 mV. Elimination of autophosphorylation-dependent CaMKII activation has a small effect on conduction velocity compared to elimination of oxidation-dependent activation.
(2.87 MB TIF)

Table S1 Model definitions and abbreviations
(0.02 MB PDF)

Table S2 Mathematical model initial conditions
(0.01 MB PDF)

Table S3 CaMKII transition rate parameters
(0.01 MB PDF)

Text S1 Model equations for NZ and BZ fiber.
(0.06 MB PDF)
