# Supplementary figures and images for: Oxidized Calmodulin Kinase II Regulates Conduction Following Myocardial Infarction: A Computational Analysis
Source: PLoS Comput Biol. 2009 Dec 4;5(12):e1000583. doi: 10.1371/journal.pcbi.1000583 (PMC2778128; doi:10.1371/journal.pcbi.1000583)

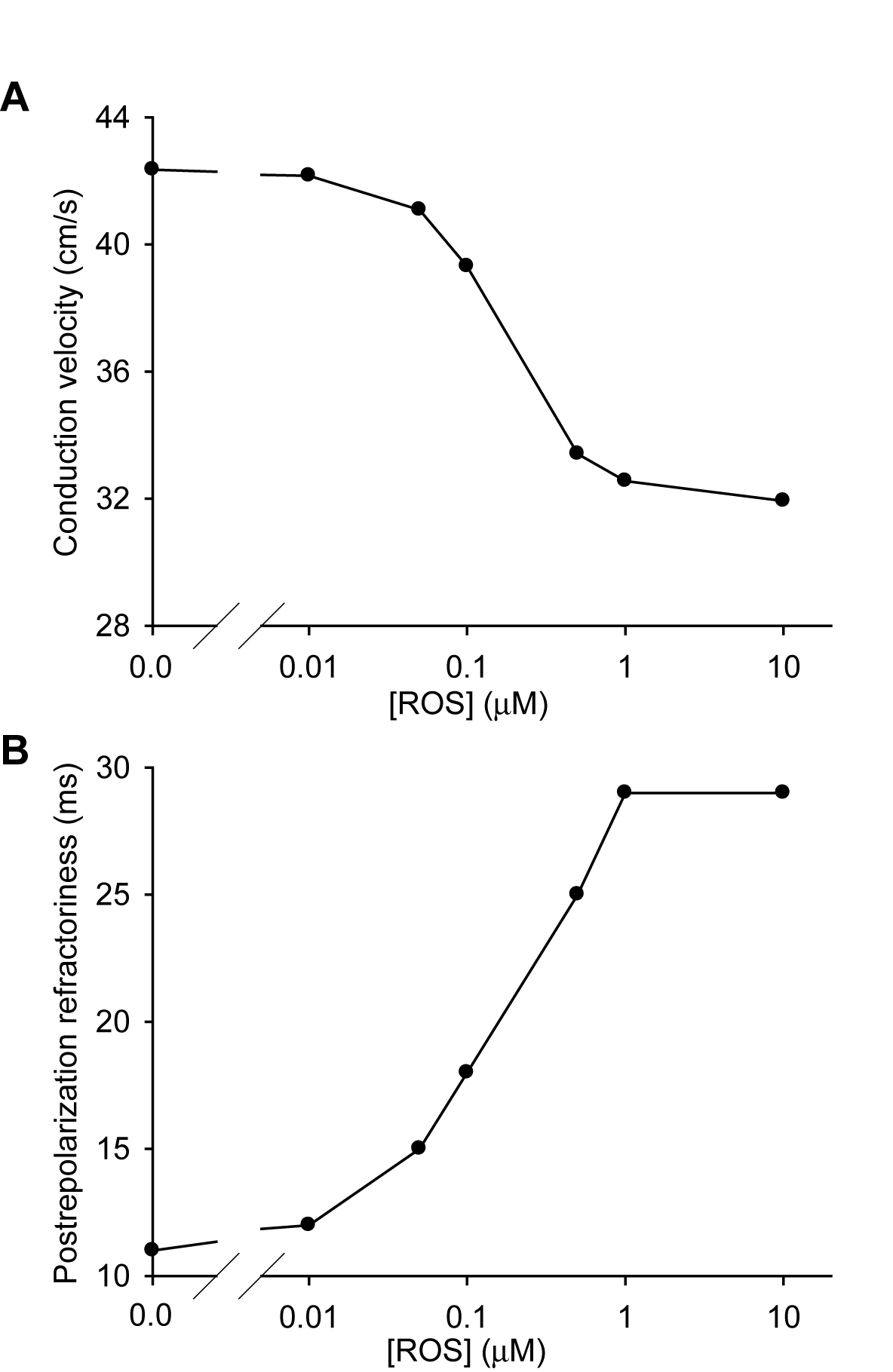

Supplement: Figure S1 — Cell excitability as a function of ROS levels in the BZ. (A) Conduction velocity and (B) refractoriness in the infarct BZ as a function of ROS concentration. Conduction velocity is determined across the middle 100 cells in the BZ fiber. Postrepolarization refractoriness is calculated as the difference between effective refractory period and action potential duration at 90% repolarization. (2.17 MB TIF) [file pcbi.1000583.s001.tif]

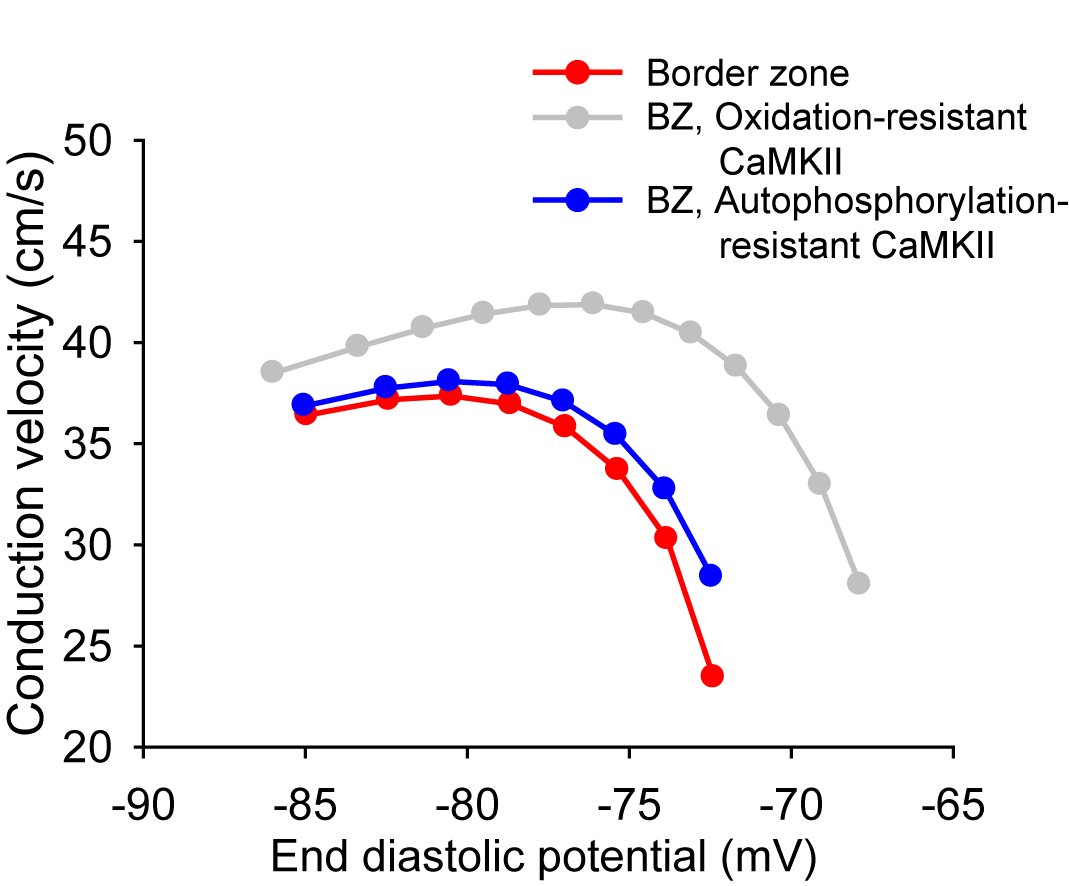

Supplement: Figure S2 — Role of autophosphorylation-dependent CaMKII activation in regulating conduction in the infarct BZ. Conduction velocity is determined across the middle 100 cells in the BZ fiber over a range of end diastolic membrane potentials (Vm,dia). [K+]o is increased incrementally from 5.4 mM to 13 mM to Vm,dia from −87 to −63 mV. Elimination of autophosphorylation-dependent CaMKII activation has a small effect on conduction velocity compared to elimination of oxidation-dependent activation. (2.87 MB TIF) [file pcbi.1000583.s002.tif]
