# Supplementary material for: Oxidized Calmodulin Kinase II Regulates Conduction Following Myocardial Infarction: A Computational Analysis
Source: PLoS Comput Biol. 2009 Dec 4;5(12):e1000583. doi: 10.1371/journal.pcbi.1000583 (PMC2778128; doi:10.1371/journal.pcbi.1000583)
Supplement: Table S1 — Model definitions and abbreviations (0.02 MB PDF) [file pcbi.1000583.s003.pdf]

Table S1. Model definitions and abbreviations.

Abbreviation	Definition
BZ	Canine epicardial border zone
NZ	Normal, remote region of infarcted heart
I_{Na}	Fast Na^+ current, µA/µF
$I_{Na,L}$	Slowly inactivating late Na^+ current, µA/µF
$I_{Ca(L)}$	Ca^{2+} current through the L-type Ca^{2+} channel, µA/µF
I_{to1}	4AP-sensitive transient outward K^+ current, µA/µF
I_{NaCa}	Na^+-Ca^{2+} exchanger, µA/µF
I_{NaK}	Na^+-K^+ pump, µA/µF
CT_{NaCl}	Na^+-Cl^- cotransporter, mmol/L per ms
CT_{KCl}	K^+-Cl^- cotransporter, mmol/L per ms
SR	Sarcoplasmic reticulum
JSR	Junctional SR
NSR	Network SR
SS	Ca^{2+} subspace
I_{rel}	Ca^{2+} release from JSR to myoplasm, mmol/L per ms
I_{up}	Ca^{2+} uptake from myoplasm to NSR, mmol/L per ms
I_{leak}	Ca^{2+} leak from JSR to myoplasm, mmol/L per ms
I_{ax}	Axial current
$I_{K,t}$	Total transmembrane K^+ current
$I_{Cl,t}$	Total transmembrane Cl^- current,
I_{stim}	Stimulus current, µA/µF
$CaMKII_{active}$	Fraction of active CaMKII subunits
f_{Bound}	Fraction of active CaMKII subunits with bound Ca^{2+}/calmodulin
f_{Phos}	Fraction of active autophosphorylated CaMKII subunits
f_{Ox}	Fraction of active oxidized CaMKII subunits
f_{OxP}	Fraction of active oxidized/autophosphorylated CaMKII subunits
f_I	Fraction of inactive CaMKII subunits
$\overline{G}_x$	Maximum conductance of channel x, mS/µF

V_m	Transmembrane potential, mV
$V_{m,dia}$	End diastolic potential, mV
$[S]_o$ and $[S]_i$	Extracellular and intracellular concentrations of ion S, respectively, mmol/L
$[Ca^{2+}]_{JSR}$	Ca^{2+} concentration in JSR, mmol/L
$[Ca^{2+}]_{NSR}$	Ca^{2+} concentration in NSR, mmol/L
$[Ca^{2+}]_{ss}$	Ca^{2+} concentration in subspace, mmol/L
$[Ca^{2+}]_{ss,t}$	Concentration of free and buffered intracellular Ca^{2+}, mmol/L
V_{SS}	Volume of SS compartment, µL.
F	Faraday constant, 96,487 C/mol
A_{Cap}	Capacitive membrane area, cm^2
R_i	Axial resistance per unit length, Ωcm, $R_{myo} + R_g / \Delta x$
R_{myo}	Myoplasmic resistivity, 150 Ωcm
R_g	Gap junction resistance, 1.5 Ωcm^2
Δx	Spatial discretization element, 0.01 cm
