# Supplementary material for: Oxidized Calmodulin Kinase II Regulates Conduction Following Myocardial Infarction: A Computational Analysis
Source: PLoS Comput Biol. 2009 Dec 4;5(12):e1000583. doi: 10.1371/journal.pcbi.1000583 (PMC2778128; doi:10.1371/journal.pcbi.1000583)
Supplement: Table S2 — Mathematical model initial conditions (0.01 MB PDF) [file pcbi.1000583.s004.pdf]

Table S2: Mathematical model initial conditions

Variable	NZ value	BZ value
V_m	-87.2162 mV	-86.1872 mV
$[Ca^{2+}]_i$	0.922820 × 10^{-4} mmol/L	1.31511 x 10^{-4} mmol/L
$[Cl^-]_i$	19.4994 mmol/L	19.1616 mmol/L
$[K^+]_i$	142.489 mmol/L	140.177 mmol/L
$[Na^+]_i$	9.88936 mmol/L	11.7219 mmol/L
$[Ca^{2+}]_{JSR}$	1.75282 mmol/L	2.42834 mmol/L
$[Ca^{2+}]_{NSR}$	1.77902 mmol/L	2.45508 mmol/L
$[Ca^{2+}]_{ss}$	0.961745 × 10^{-4} mmol/L	1.32944 x 10^{-4} mmol/L
$CaMK_{active}$	3.64721 x 10^{-4}	2.39046 x 10^{-5}
f_{Bound}	4.86283 x 10^{-4}	3.35299 x 10^{-6}
f_{Phos}	8.38259 x 10^{-9}	5.48067 x 10^{-12}
f_{Ox}	0	4.27796 x 10^{-5}
f_{OxP}	0	6.99266 x 10^{-11}
f_I	0.999514	0.999954
$[Ca^{2+}]_o$	1.8 mmol/L	1.8 mmol/L
$[Cl^-]_o$	100 mmol/L	100 mmol/L
$[K^+]_o$	5.4 mmol/L	5.4 mmol/L
$[Na^+]_o$	140 mmol/L	140 mmol/L
