# Supplementary material for: Oxidized Calmodulin Kinase II Regulates Conduction Following Myocardial Infarction: A Computational Analysis
Source: PLoS Comput Biol. 2009 Dec 4;5(12):e1000583. doi: 10.1371/journal.pcbi.1000583 (PMC2778128; doi:10.1371/journal.pcbi.1000583)
Supplement: Table S3 — CaMKII transition rate parameters (0.01 MB PDF) [file pcbi.1000583.s005.pdf]

Table S3. CaMKII transition rate parameters.

Parameter	Value	References
k_{IB}	246 mM^{-1} ms^{-1}	Meyer et al., Gaertner et al.*
k_{BI}	0.0022 ms^{-1}	Meyer et al.
k_{BOx}	2.91 x 10^{-4} μM^{-1} ms^{-1}	Erickson et al.*
k_{OxB}	2.23 x 10^{-5} ms^{-1}	-
k_{PB}	0.00003 ms^{-1}	Hund et al.
k_{POxP}	k_{BOx}	-
k_{OxPP}	K_{OxB}	-
k_{OxPOx}	k_{PB}	-

*Derived
