# Supplementary material for: Oxidized Calmodulin Kinase II Regulates Conduction Following Myocardial Infarction: A Computational Analysis
Source: PLoS Comput Biol. 2009 Dec 4;5(12):e1000583. doi: 10.1371/journal.pcbi.1000583 (PMC2778128; doi:10.1371/journal.pcbi.1000583)
Supplement: Text S1 — Model equations for NZ and BZ fiber. (0.06 MB PDF) [file pcbi.1000583.s006.pdf]

Supplementary text
Christensen et al. Oxidized CaMKII regulates conduction. *PLoS Comput Biol*.

Model equations for NZ and BZ fiber

Complete HRd control (NZ) and BZ model equations may be found in previous publications [1,2]. Here we provide equations and parameters that differ from the original models.

A. Transmembrane potential, V_m

Action potential propagation is simulated by discretizing the equation describing axial current flow along the fiber and solving numerically using the Crank-Nicholson implicit method.

$$-\frac{\partial I_{ax}}{\partial x} = \frac{a}{2R_i} \cdot \frac{\partial^2 V_m(x,t)}{\partial x^2} = C_m \frac{\partial V_m(x,t)}{\partial t} + \sum I_{ion}$$

The ends of the fiber are assumed to be sealed (no-flow boundary conditions, $\partial V / \partial x = 0$ at first and last element of the fiber). To preserve conservation (and to simulate propagation using the algebraic method), axial current is assumed to carry K^+ ions (I_m is added to the total K^+ current) as previously recommended [3,4].

B. CaMKII activity

CaMKII activity is based on the model of Dupont et al.[5] with modifications to include oxidative activation as described recently [6]. Oxidation occurs from the Ca^{2+}/calmodulin bound state, as observed experimentally [6]. Also, the model includes a single autophosphorylation state rather than multiple states used in previous models [7-9]. Inclusion of an additional autonomous (phosphorylated but no bound Ca^{2+}/CaM) state was found to have no impact on model behavior (state occupancy < 0.001%, not shown) and was therefore not included in the final model. We also include a state where a subunit is both oxidized and phosphorylated (C_{OxP}). Rate constants are taken from the literature [2,8-11] or are chosen to fit experimental data (see Figure 3 and Table S3). Following the formulation of Dupont et al., the autophosphorylation rate is a phenomenological function of total CaMKII activity chosen to fit experimental dependence of autonomous activity on calmodulin [7]. The relative activity of the Ca^{2+}/calmodulin bound active state (C_{Bound}) is taken from Dupont et al. to be less than the relative activity of the autophosphorylated state (C_{Phos}) [5]. We assume relative activity of 50% for oxidized CaMKII (C_{Ox}) consistent with experiment [6].

Our model assumes that only saturated CaM (4 Ca^{2+} bound to CaM) activates CaMKII. However, a previous modeling study has shown that nonsaturated calmodulin (2 Ca^{2+} bound to CaM) binding to CaMKII with subsequent recruitment of 2 Ca^{2+} is an alternative (perhaps preferable) pathway for kinase activation [9]. To determine the response of our model to nonsaturated CaM, we incorporated a more detailed and well-validated representation of Ca^{2+} binding to CaM includes apoCaM, nonsaturated CaM ($2Ca^{2+}$/CaM), and saturated CaM ($4Ca^{2+}$/CaM) [9]. In this model, $2Ca^{2+}$/CaM binding to CaMKII may activate the kinase only after subsequent recruitment of 2 Ca^{2+}. This modified pathway was incorporated into our BZ model which was paced to steady-state at a cycle length of 500 ms. We also performed simulations using a model where only

saturated CaM ($4Ca^{2+}$/CaM but not $2Ca^{2+}$/CaM) could bind to CaMKII. Importantly, 1 μM ROS resulted in a similar increase in CaMKII activity independent of whether or not $2Ca^{2+}$/CaM could bind to CaMKII (21% and 18% maximal activity, respectively, compared to 0.72% with [ROS] = 0 μM, not shown). Thus, we have opted to retain our simplified scheme of Ca^{2+}/CaM binding for the remaining simulations.

$$\frac{df_{Bound}}{dt} = k_{IB} \cdot calm \cdot f_I + k_{PB} \cdot f_{Phos} + k_{OxB} \cdot f_{Ox} - (k_{BI} + k_{BOx} \cdot ROS) \cdot f_{Bound} - k_A \cdot f_{Bound}$$

$$calm = \overline{calm} \cdot (1 + (0.005/[Ca^{2+}]_{ss})^h)^{-1}$$

$\overline{calm} = 60$ nM is free calmodulin concentration [12]; $h = 4$

$$\frac{df_{Phos}}{dt} = k_A \cdot f_{Bound} + k_{OxPP} \cdot f_{OxP} - (k_{PB} + k_{POxP} \cdot ROS) \cdot f_{Phos}$$

$$k_A = k_{BI} \cdot T_{CaMK} / (T_{CaMK} + 0.01851)$$

$$T_{CaMK} = k_{BI} / k_{IB} \cdot (1/(f_{Bound} + f_{Phos} + f_{Ox} + f_{OxP}) - 1)^{-1}$$

$$\frac{df_{Ox}}{dt} = k_{BOx} \cdot ROS \cdot f_{Bound} + k_{OxPOx} \cdot f_{OxP} - (k_{OxB} + k_A) \cdot f_{Ox}$$

$$\frac{df_{OxP}}{dt} = k_A \cdot f_{Ox} + k_{POxP} \cdot ROS \cdot f_{Phos} - (k_{OxPP} + k_{OxPOx}) \cdot f_{OxP}$$

Unless otherwise stated, *ROS* = 1.0 μM for BZ model and 0.0 μM for NZ.

$$f_I = 1 - f_{Bound} - f_{Phos} - f_{Ox} - f_{OxP}$$

$$CaMKII_{active} = C_{Bound} \cdot f_{Bound} + C_{Phos} \cdot f_{Phos} + C_{Ox} \cdot f_{Ox} + C_{OxP} \cdot f_{OxP}$$

$$C_{Bound} = 0.75;\ C_{Phos} = C_{OxP} = 1.0;\ C_{Ox} = 0.5$$

C. L-type Ca^{2+} current, $I_{Ca,L}$

$K_{m,CaMK} = 0.2$; $h_{\infty,f} = h_{\infty,fca} = 2.0$

D. SR Ca^{2+} release, I_{rel}

$K_{m,CaMK} = 0.2$

E. Fast Na^+ current, I_{Na}

$K_{m,CaMK} = 0.3$

F. Late Na^+ current, $I_{Na,l}$

$K_{m,CaMK} = 0.3$

G. K^+-Cl^- cotransporter, CT_{KCl}

We adopt conductance values for CT_{KCL} and CT_{NaCl}, used by Decker et al. [13].

$$\overline{CT}_{KCl} = 1.77 \times 10^{-5} \text{ mmol/L per ms}$$

H. Na^+-Cl^- cotransporter, CT_{NaCl}

$$\overline{CT}_{NaCl} = 2.46108 \times 10^{-5}$$

Supplementary text

Christensen et al. Oxidized CaMKII regulates conduction. *PLoS Comput Biol*.

I. Na^{+}/K^{+} ATPase, I_{NaK}

To maintain resting $[K^{+}]_i$ similar to original HRd model, the following value is used for the maximal pump current.

$$\bar{I}_{NaK} = 0.93\ \mathrm{mS/\mu F}$$

References

1. Hund TJ, Rudy Y (2004) Rate dependence and regulation of action potential and calcium transient in a canine cardiac ventricular cell model. Circulation 110: 3168-3174.

2. Hund TJ, Decker KF, Kanter E, Mohler PJ, Boyden PA, et al. (2008) Role of activated CaMKII in abnormal calcium homeostasis and I_{Na} remodeling after myocardial infarction: Insights from mathematical modeling. J Mol Cell Cardiol 45: 420-428.

3. Ten Tusscher KH, Noble D, Noble PJ, Panfilov AV (2003) A model for human ventricular tissue. Am J Physiol Heart Circ Physiol 286: H1573-1589.

4. Hund TJ, Kucera JP, Otani NF, Rudy Y (2001) Ionic charge conservation and long-term steady state in the Luo-Rudy dynamic model of the cardiac cell. Biophys J 81: 3324-3331.

5. Dupont G, Houart G, De Koninck P (2003) Sensitivity of CaM kinase II to the frequency of Ca^{2+} oscillations: a simple model. Cell calcium 34: 485-497.

6. Erickson JR, Joiner ML, Guan X, Kutschke W, Yang J, et al. (2008) A dynamic pathway for calcium-independent activation of CaMKII by methionine oxidation. Cell 133: 462-474.

7. Dupont E, Matsushita T, Kaba RA, Vozzi C, Coppen SR, et al. (2001) Altered connexin expression in human congestive heart failure. J Mol Cell Cardiol 33: 359-371.

8. Hashambhoy YL, Winslow RL, Greenstein JL (2009) CaMKII-induced shift in modal gating explains L-type Ca^{2+} current facilitation: a modeling study. Biophys J 96: 1770-1785.

9. Saucerman JJ, Bers DM (2008) Calmodulin mediates differential sensitivity of CaMKII and calcineurin to local Ca^{2+} in cardiac myocytes. Biophys J 95: 4597-4612.

10. Gaertner TR, Kolodziej SJ, Wang D, Kobayashi R, Koomen JM, et al. (2004) Comparative analyses of the three-dimensional structures and enzymatic properties of α, β, γ and δ isoforms of Ca^{2+}-calmodulin-dependent protein kinase II. J Biol Chem 279: 12484-12494.

11. Meyer T, Hanson PI, Stryer L, Schulman H (1992) Calmodulin trapping by calcium-calmodulin-dependent protein kinase. Science 256: 1199-1202.

12. Wu X, Bers DM (2007) Free and bound intracellular calmodulin measurements in cardiac myocytes. Cell Calcium 41: 353-364.

13. Decker KF, Heijman J, Silva JR, Hund TJ, Rudy Y (2009) Properties and ionic mechanisms of action potential adaptation, restitution and accommodation in canine epicardium. Am J Physiol Heart Circ Physiol.
